# Identifying the prevalence and predictors of suicidal behaviours for indigenous males in custody

**DOI:** 10.1186/s12889-018-6074-5

**Published:** 2018-10-04

**Authors:** Stephane M Shepherd, Benjamin Spivak, Kerry Arabena, Yin Paradies

**Affiliations:** 1 Centre for Forensic Behavioural Science, Swinburne University of Technology, 505 Hoddle Street, Clifton Hill 3068, Melbourne, VIC 9947 2600 Australia; 2 National Centre for Cultural Competence, University of Sydney, Sydney, Australia; 3 Indigenous Health Equity Unit, Melbourne School of Population Health, University of Melbourne, Melbourne, Australia; 4 Alfred Deakin Research Institute for Citizenship and Globalisation, Deakin University, Geelong, Australia

**Keywords:** Indigenous population, Suicide, Prison, Mental illness, Aboriginal and Torres Strait islander health

## Abstract

**Background:**

High rates of suicidal behaviours among Indigenous Australians have been documented. Justice-involved individuals are also at a higher risk for engaging in suicidal behaviours. This study sought to ascertain the prevalence and correlates of suicidal behaviours for 107 Indigenous adult males in custody in Victoria, Australia.

**Methods:**

Participants undertook a structured interview comprising a psychiatric assessment. Information on suicidal behaviours (ideation and attempts), socio-demographics, environmental stressors, negative life events and mental health was obtained.

**Results:**

A high proportion of Indigenous males in custody experienced lifetime suicidal ideation (63.7%) and over one-half had attempted suicide (54.5%). A smaller, yet significant number of participants experienced ideation over the past 12 months (27.9%). Having a loved one pass away within the past 12 months predicted recent ideation; lifetime ideation and a diagnosis of Post-Traumatic Stress Disorder predicted a lifetime suicide attempt.

**Conclusions:**

The prevalence of suicidal behaviours among Indigenous people in custody is remarkably high. Correlates of suicidal behaviours for Indigenous people in custody in this study likely manifest in the community, denoting an urgent public health response. Prevention must begin in communities at-risk for suicidal behaviours. The development of low intensity mental health service infrastructure in communities to promote awareness and provide accessible, least restrictive support and treatment is necessary. Correctional institutions must also continue to improve custodial suicide prevention and management initiatives.

## Background

In a given year, almost 800,000 people die by suicide worldwide [1]. In Australia, suicide is the leading cause of death for people aged between 15 and 44 years [2]. Indigenous Australians (Aboriginal and Torres Strait Islanders) are at a higher risk for suicide compared to non-Indigenous Australians. Almost 6% of Indigenous Australian deaths are the result of suicide compared with less than 2% of non-Indigenous Australian deaths [2]. This disparity is more pronounced in early adulthood [2, 3], and among young people under 18 years [2]. The higher rate of suicide among Indigenous Australians has been linked to socio-economic disadvantage, racism, cultural disconnection, alienation and exposure to a concert of traumatic stressors and negative life events (e.g., incarceration, frequent preventable deaths of family and friends, child removal, family breakdown, sexual/physical abuse) [4–15]. These factors are often associated with other correlates of suicidal behaviours such as psychological distress, mental disorder, substance abuse, homelessness and ‘social and emotional wellbeing’ [5–7, 9, 13, 16–21], all of which are intensified in the absence of community support networks. Social and emotional wellbeing (SEWB) is an Indigenous Australian concept of health that encompasses physical, psychological, cultural, spiritual, familial and community dimensions [22]. The holistic nature of SEWB is believed to diverge from biomedical characterisations of illness which are described as having a more individualized emphasis [22]. The above risk factors, including low SEWB have been associated with criminal justice system contact [23–25]. Indigenous Australians are disproportionately incarcerated in every state and territory [26], which contributes to cycles of disadvantage, and risk for suicidal behaviours.

Rates of suicide are higher for adults who are incarcerated compared to the general community [27]. Detainees often present with numerous complex social and clinical needs [28, 29] which increase their risk for re-offending and also their risk for suicide [9, 30–32]. The custodial environment may increase levels of psychological distress, emotional breakdown, frustration and vulnerability to victimization [33]. As such, suicide is often the most common cause of death within correctional institutions after deaths due to natural causes [34]. Moreover, the risk for suicide is elevated during the immediate post-release period [35–37]. This presents a heightened public health concern for some Indigenous Australians given the higher rates of incarceration in addition to higher rates of suicide in the broader community [3]. It is still unclear however, whether Indigenous Australians in custody are at a higher risk for suicide compared to non-Indigenous prisoners. The 1991 Royal Commission into Aboriginal Deaths in Custody discovered that rates of death (suicide or otherwise) are similar for Aboriginal and non-Aboriginal people in custody [8]. More contemporary reports indicate that rates of Indigenous deaths in custody (including self-inflicted deaths) have not increased alongside growing Indigenous imprisonment rates [34, 38]. In fact, death in custody rates for Indigenous prisoners may have decreased and now occur at lower rates compared to non-Indigenous prisoners [34].

Evidence suggests that Australian prison suicides have declined over time [38], presumably due to improved preventive initiatives in custody [39]. However, many inmates possess histories of suicidal behaviour and arrive in custody presenting with risk factors for suicide. It is estimated that around one-third of Australian prisoners have experienced suicidal ideation in their lives and approximately one-fifth have attempted suicide [40]. Higher percentages of ideation and attempted suicide have been identified among Indigenous prisoners [9, 41]. Despite this finding, risk for suicide on release from custody remains unchanged for Indigenous people [35]. However, this risk is still significantly greater than for those in the general population [14]. As such, there is a need to gain a better understanding of the drivers of suicidal behaviour for Indigenous people who find themselves in custody. Prior work has investigated the correlates of suicidal ideation and suicide attempts for the wider Australian prison population [9]. Yet no research has sought to identify correlates for Indigenous prisoners specifically. It is important for correctional and community health services to be able to identify and address the needs of justice-involved Indigenous people at-risk for suicide. This group potentially faces both multiple and unique factors that increase their risk for suicidal behaviours in custody and when transitioning back to the community.

This study aims to identify the prevalence and correlates of suicidal ideation and suicide attempts for a cohort of Indigenous people in custody in the Australian state of Victoria. We expect mental health factors, negative life events and custodial history to be associated with suicidal behaviours. Histories of psychiatric disorder and life stressors have previously been correlated with suicidal ideation in Australian prison populations. We also expect ideation to be associated with life-time suicide attempts, reflecting prior forensic and public health research.

## Method

### Recruitment setting

All remanded and sentenced male Aboriginal and Torres Strait Islander prisoners over 18 years from 11 regional and metropolitan prisons in the state of Victoria were approached to participate in the study. Participants were required to have their Aboriginal and Torres Strait Islander status formally registered with prison services. There was no recruitment from one correctional centre due to insufficient numbers of Indigenous prisoners. Participants placed in management at the time of interviews were not eligible to participate in the study. Over the course of recruitment, two prisoners declined to participate in the study once the researcher had explained the study to them.

### Materials

#### Structured interview

A structured interview was conducted with each participant to obtain data on suicidal behaviours, mental health, environmental stressors and socio-demographic information. This encompassed information pertaining to a participant’s area of residence prior to custody (rural and remote or urban), custodial status (sentenced or remanded), the total number of episodes in custody as an adult and the total number of days spent in custody for the current sentence or remand period.

#### Suicidal behaviours

Participants were asked to indicate (Yes/No) whether they had ever attempted suicide, whether they had ever had thoughts of suicide (ideation), and whether they had thoughts of suicide in the 12 months prior to the interview. In addition, participants were also asked about the locations (custody/community) in which they had attempted suicide or had suicidal thoughts.

Participants were also asked if a friend or relative had ever died by suicide and if a friend or relative had passed away (any cause) within the previous 12 months.

#### MINI international neuropsychiatric interview (MINI)

The Mini International Neuropsychiatric Interview (MINI) is a structured diagnostic interview designed by psychiatrists to assess the presence of psychiatric disorders as defined in the Diagnostic and Statistical Manual of Mental Disorders (DSM-IV) and the ICD-10 (International Classification of Diseases and Related Health Problems). The MINI comprises a series of yes/no questions across a number of clinical domains (e.G. *major* Depressive Episode, Dysthymia, Social Phobia). The presence of a disorder is indicated based on particular patterns of responses within each domain. The MINI has demonstrated concordance with other structured clinical interviews [42] and acceptable agreement with clinical diagnoses [43]. The MINI has previously been employed with Australian Indigenous populations [44].

#### Mood disorder

For the purposes of the present study, all MINI items related to a lifetime diagnosis of a mood disorder were included (i.e., presence of major depressive disorder, major depressive disorder with psychotic features, Bipolar I and II, Bipolar NOS, Bipolar I with psychotic features and Mood disorder NOS).

#### Substance use disorder

All MINI items indicating the presence of a substance use disorder were included (i.e., all items indicating the presence of alcohol dependence, alcohol abuse, substance dependence and substance abuse).

#### Anxiety disorder

MINI items indicating a diagnosis of social phobia, obsessive compulsive disorder, panic disorder and generalised anxiety disorder were collapsed into a single variable indicating the presence or absence of an anxiety disorder.

#### Psychotic disorder

MINI items indicating a diagnosis of psychotic disorder, psychotic disorder due to medical condition, substance induced psychotic disorder, brief psychotic disorder, schizophrenia, schizoaffective disorder and schizophreniform disorder were collapsed into a single variable indicating the presence or absence of a psychotic disorder.

#### Post-traumatic stress disorder

A diagnosis of PTSD based on the MINI was used as a variable to indicate the presence or absence of PTSD.

### Procedure

The data for this analysis comprised the Koori Prisoner Mental Health and Cognitive Function Study (KPMHS) database sample. The KPMHS was conducted by the Centre for Forensic Behavioural Science under contract from the Victorian Department of Justice to investigate the mental health needs of Koori prisoners. Ethical approval to utilise the database was obtained from the Victorian Department of Justice Human Research Ethics Committee and Swinburne University Human Research Ethics Committee. The KPMHS study was overseen by a steering committee which included representation from the Victorian Aboriginal Community Controlled Health Organisation, the Victorian Aboriginal Legal Service and the Koori Justice Unit (Victorian Department of Justice).

As part of the KPMHS, participants undertook a structured interview and were administered a battery of mental health questionnaires in custody between January and October 2012. The development of the structured interview with Koori prisoners involved close consultation with the steering committee and advisory group. It was also reviewed by an Aboriginal psychologist with research expertise, as well as an Aboriginal psychologist with neuropsychological experience and a clinical neuropsychologist.

The interview was conducted by two mental health practitioners, one of whom was Aboriginal. Clients were initially informed of the study by Aboriginal Wellbeing or Liaison officers who provide support for Indigenous prisoners and cultural advice for prison staff. Aboriginal Wellbeing/Liaison Officers at each prison briefly informed participants of the details of the study. Those prisoners interested in participating in the study then met with the interviewers who provided them with an explanatory statement. The Aboriginal and Torres Strait Islander research officer verbally reviewed the explanatory statement with the prisoner and provided an opportunity for the prisoner to ask questions. Prisoners who wished to take part were asked to sign a consent form acknowledging their understanding of the study. All interviews were conducted in private rooms visible (though not audible) to custodial staff. Participation in the study was voluntary and participants could choose not to answer any questions, or terminate the interview at any time, if desired.

### Statistical approach

The prevalence of suicidal ideation and suicide attempts across the lifetime, and suicidal ideation 12 months prior to baseline (time of interview) was estimated by calculating the proportion of participants who reported suicidal ideation and attempts in each of these categories. Confidence intervals were also calculated for the proportions of participants in each category. The median length of time in custody was examined between participants who reported suicidal ideation and those who did not report suicidal ideation in the 12 months prior to baseline. A Wilcoxon test was used to examine the average difference between those reporting suicidal ideation over the past 12 months and those who did not. Following the examination of time in custody, the custody status of participants was examined by reporting the proportion of participants who had been sentenced compared to the proportion who had been remanded.

To examine the correlates of suicidal ideation and suicide attempts, three multivariate logistic regression models were utilised. The model for lifetime suicidal ideation contained total number of times in custody, self-report of a close friend or relative dying by suicide, lifetime diagnosis of mood disorder, lifetime diagnosis of substance use disorder, lifetime diagnosis of anxiety disorder, lifetime diagnosis of a psychotic disorder, lifetime diagnosis of PTSD and the participant’s region of residence outside of custody (rural/remote vs city).

The model for 12 month suicidal ideation was similar. However, rather than examining the total number of times in custody, this variable was replaced by the total amount of time in custody on the present occasion. This would help determine whether greater exposure to the prison experience is contributing to ideation. The report of a close friend or relative dying by suicide was replaced with a self-report variable indicating whether or not a friend or relative had passed away in the previous 12 months.

The same variables were used in the model for lifetime suicidal ideation. The only difference being that lifetime ideation was included as a variable in the lifetime attempts model. Odds ratios and associated 95% confidence intervals were presented for each variable adjusted for all other variables in the model and also in unadjusted form.

## Results

The sample included 107 Indigenous males in custody. The sample size is representative of Indigenous prisoners in Victoria, which possesses the lowest proportion of Indigenous people in custody nationwide. The mean age of participants was 34.2 (*SD* = 10.4, range: 18–62) years. The sample comprised 100 (93.5%) participants who identified as Aboriginal and 5 (4.5%) who identified as Torres Strait Islander. A minority of participants were in custody on remand (*n* = 30, 28.0%), the remainder were undertaking a custodial sentence (*n* = 75, 70%). Data on specific Indigenous heritage (Aboriginal and/or Torres Strait Islander) and custody status was not available for two of the participants.

### Prevalence of suicidal ideation and suicide attempts

Prevalence data for each of the outcome assessments used are presented in Table 1.

**Table 1:** Proportion of participants reporting self-harm behaviour

	n/N	%	(95% CI)
Suicidal ideation in previous 12 months	24/86	27.9	19–39
Lifetime suicidal ideation	65/102	63.7	54–73
Lifetime suicide attempts	49/90	54.4	44–65

Suicidal ideation followed a decreasing pattern in relation to the time period examined, with the highest proportion reported for lifetime suicidal ideation, followed by ideation in the previous 12 months. Approximately 74% (*n* = 48) of participants who reported lifetime suicidal ideation also reported a suicide attempt.

Participants who reported lifetime suicidal ideation and lifetime suicide attempts were asked to provide follow up data relating to locations in which suicide attempts had been made and where thoughts of suicide had been worst. Of the participants that reported lifetime suicidal ideation and responded to the location question (*n* = 61), the majority (*n* = 37, 61%) reported that their worst thoughts of suicide occurred in the community, a smaller proportion identified custodial settings (*n* = 16, 26%), eight participants (13%) reported that their worst thoughts of suicide occurred in both community and custodial settings. The location of suicide attempts followed a similar pattern, with the majority of those who responded to the question (*n* = 47) reporting that their attempts took place in the community (*n* = 34, 72%), with far fewer reporting suicide attempts in custodial settings (n = 4, 9%), nine participants (19%) reported that suicide attempts occurred in both community and custodial settings.

All but two participants reported the number of times that they had served custodial sentences as an adult. The median number of custodial sentences was 4 (*IQR* = 2–6, *range* = 1–45) custodial sentences. The median number of sentences previously served was not significantly different for participants who reported suicidal ideation in the past 12 months (*Median* = 3, *IQR* = 1–5, *range* = 1–20) compared with participants who reported no suicidal ideation in the same period (*Median* = 4, *IQR* = 1–6, *range* = 1–4), *W* = 770, *p* = .7. Similarly, participants reporting lifetime suicidal ideation (*Median* = 4, *IQR* = 2–5, *range* = 1–45) did not significantly differ from participants who reported no suicidal ideation (*Median* = 5, *IQR* = 1–6, *range* = 1–15), *W* = 1200, *p* = .6. Finally, participants reporting lifetime suicide attempts (*Median* = 4, IQR = 1–6, *range* = 1–45) did not differ significantly from participants who reported no suicide attempts (*Median* = 3.5, *IQR* = 2–6, *range* = 1–20) in terms of the number of times in custody as an adult, *W* = 980, *p* > .99.

The median number of days spent in the most recent time in custody was then examined between participants who had suicidal ideation in the past 12 months (*Median* = 9.5, *IQR* = 5–18.75, *range* = 1–180) compared to those who had not reported suicidal ideation in the same time period (*Median* = 17, *IQR* = 6–36, *range* = 0.6–114). The average time was slightly higher among those reporting no suicidal ideation. However, Wilcoxon’s test indicated that the differences were not significant, *W* = 810, *p* = .4.

Finally, custody status was examined between participants who reported suicidal ideation in the past 12 months. Among those reporting suicidal ideation, the majority were serving custodial sentences (*n* = 17, 71%) rather than on remand (*n* = 7, 29%). Those who reported no suicidal ideation over the same period were mostly serving custodial sentences (*n* = 42, 70%) with a minority on remand (*n* = 18, 30%).

### Correlates of suicidal ideation and suicide attempts

Correlates of lifetime suicide ideation, suicidal ideation in the 12 months prior to baseline, and lifetime suicide attempts were examined through the use of three multivariable logistic regression models. Hosmer and Lemeshow tests for all three models were not significant (Lifetime Suicidal Ideation: *χ*^*2*^ [8] = 6.2, *p* = .6; 12 Month Suicidal Ideation: *χ*^*2*^ [8]= 8.3, *p* = .4; Suicide Attempts: *χ*^*2*^ [8]= 8.7, *p* = .4) indicating a lack of evidence for poor model fit.

The total effect size for the lifetime suicidal ideation model was evaluated using Nagelkerke’s R^2^, a coefficient which ranges from 0 (equivalent to no predictive power) to 1 (with 1 equivalent to perfect prediction), the R^2^ for the model was 0.24. Results are presented in Table 2.

**Table 2:** Correlates of lifetime suicidal ideation

	No suicidal ideation (*N* = 35)			Suicidal ideation (*N* = 61)				
	n	%	(95% CI)	n	%	(95% CI)	OR (95%CI)	AOR (95%CI)^a^
Number of times in custody	–	–	–	–	–	–	1.0 (0.9–1.1)	1.0 (0.9–1.2)
No suicide of close friend or relative	14	40	24–58	17	28	18–41	–	–
Suicide of close friend or relative	21	60	42–76	44	72	59–82	1.7 (0.7–4.2)	1.2 (0.4–3.1)
Non rural/remote	15	43	27–60	27	44	32–57	–	–
Rural/remote	20	57	40–73	34	56	43–68	0.9 (0.4–2.2)	1.2 (0.5–3.4)
No lifetime mood disorder	24	69	51–83	33	54	41–67	–	–
Lifetime mood disorder	11	31	17–49	28	46	33–59	1.9 (0.8–4.6)	2.1 (0.8–6.1)
No lifetime substance use disorder	11	31	17–49	18	30	19–43	–	–
Lifetime substance use disorder	24	69	51–83	43	70	57–81	1.1 (0.4–2.7)	0.8 (0.3–2.0)
No lifetime anxiety disorder	30	86	69–95	43	70	57–81	–	–
Lifetime anxiety disorder	5	14	5–31	18	30	19–43	2.5 (0.9–8.3)	1.7 (0.5–6.0)
No lifetime psychotic disorder	33	94	79–99	52	85	73–93	–	–
Lifetime psychotic disorder	2	6	1–21	9	15	7–27	2.9 (0.7–19.5)	3.5 (0.7–26.7)
No lifetime PTSD	33	94	79–99	48	79	66– – 88	–	–
Lifetime PTSD	2	6	1–21	13	21	12–34	4.5 (1.1–29.8)	4.1 (0.9–29.7)

^a^Odds ratios are adjusted for all variables listed in the model

The results of the model indicate that none of the variables was significantly associated with the outcome. The results of the 12 month suicidal ideation model are presented in Table 3.

**Table 3:** Correlates of suicidal ideation in previous 12 months

	No suicidal ideation (*N* = 60)			Suicidal ideation (*N* = 22)				
	N	%	(95% CI)	n	%	(95% CI)	OR (95%CI)	AOR (95%CI)^a^
Current length of time spent in custody	–	–	–	–	–	–	1.0 (0.99–1.02)	1.0 (0.99–1.02)
No close friend or relative passed away in previous 12 months	34	57	43–69	5	23	9–46	–	–
Close friend or relative passed away in previous 12 months	26	43	31–57	17	77	54–91	4.4 (1.5–15.0)**	5.3 (1.6–20.9) **
No diagnosis of PTSD	52	87	75–94	16	73	50–88	–	–
Diagnosis of PTSD	8	13	6–25	6	27	12–50	2.4 (0.7–8.1)	1.9 (0.5–7.8)
No lifetime mood disorder	38	63	50–75	11	50	31–69	–	–
Lifetime mood disorder	22	37	25–50	11	50	31–69	1.7 (0.6–4.7)	2.2 (0.7–7.9)
No lifetime substance use disorder	18	30	19–43	5	23	9–46	–	–
Lifetime substance use disorder	42	70	57–81	17	77	54–91	1.5 (0.5–5.0)	2.0 (0.5–9.1)
No anxiety disorder	48	80	67–89	14	64	40–82	–	–
Anxiety disorder	12	20	11–33	8	36	18–59	2.3 (0.8–6.7)	2.3 (0.6–8.6)
No psychotic disorder	53	88	77–95	19	86	64–96	–	–
Psychotic disorder	7	12	5–23	3	14	4–36	1.2 (0.2–4.8)	0.7 (0.1–3.7)
Non rural/remote	28	47	34–60	8	36	18–59	–	–
Rural/remote	32	53	40–66	14	64	40–82	1.5 (0.6–4.3)	2.8 (0.8–11.2)

^a^Odds ratios are adjusted for all variables listed in the model

**p < .01

Nagelkerke’s R^2^ for the total model was 0.34, indicating moderate explanatory power. The reporting of a close friend or relative passing away in the 12 months prior to baseline was significantly associated with suicidal ideation over the previous 12 months (adjusted odds ratio = 5.3, 95% CI = 1.6–20.9, *p* = .009). The results of the suicide attempts model are presented in Table 4.

**Table 4:** Correlates of suicide attempts

	No suicide attempt(*N* = 40)			Suicide attempt (*N* = 45)				
	N	%	(95% CI)	N	%	(95% CI)	OR (95%CI)	AOR (95%CI)^a^
Number of times in custody	–	–	–	–	–	–	1.0 (0.96–1.1)	1.0 (0.96–1.2)
No suicide of close friend or relative	14	35	21–52	13	29	17–45	–	–
Suicide of close friend or relative	26	65	48–79	32	71	55–83	1.3 (0.5–3.3)	0.6 (0.1–2.4)
No diagnosis of PTSD	38	95	82–99	33	73	58–85	–	–
Diagnosis of PTSD	2	5	1–18	12	27	15–42	6.9 (1.7–46.5)*	5.1 (0.8–62.0)
No lifetime mood disorder	28	70	53–83	22	49	34–64	–	–
Lifetime mood disorder	12	30	17–47	23	51	36–66	2.4 (1.0–6.1)	2.7 (0.7–11.2)
No lifetime substance use disorder	15	38	23–54	10	22	12–37	–	–
Lifetime substance use disorder	25	62	46–77	35	78	63–88	2.1 (0.8–5.6)	2.7 (0.7–10.8)
No anxiety disorder	34	85	69–94	30	67	51–80	–	–
Anxiety disorder	6	15	6–31	15	33	20–49	2.8 (1.0–8.8)	1.5 (0.3–7.4)
No psychotic disorder	37	93	79–98	38	84	70–93	–	–
Psychotic disorder	3	7	2–21	7	16	7–30	2.3 (0.6–11.2)	1.4 (0.2–12.3)
No lifetime suicidal ideation	23	58	41–73	1	2	0–13	–	–
Suicidal ideation	17	43	27–59	44	98	87–100	59.5 (11.2–1107.1)***	58.2 (9.6–1159.6)***
Non rural/remote	16	40	25–57	22	49	34–64	–	–
Rural/remote	24	60	43–75	23	51	36–66	0.7 (0.3–1.6)	0.7 (0.2–2.8)

^a^Odds ratios are adjusted for all variables listed in the model

*p < .05; ***p < .001

Nagelkerke’s R^2^ for the total model was 0.62, indicating good explanatory power. Suicidal ideation (AOR = 58.2, 95%CI = 9.6–1159.6, *p* < .001) was significantly associated suicide attempts. No other variables were significantly associated with the outcome when adjusting for other variables. A diagnosis of PTSD was associated with suicidal attempts univariately (OR = 6.9, 95%CI = 1.7–46.5, *p* = .02).

## Discussion

This is the first study to identify both the prevalence and correlates of suicidal behaviours for a cohort of Indigenous males in custody. The motivations for this analysis were underpinned by three key phenomena. First, offenders regularly possess numerous socio-historical and clinical factors that increase their risk for suicide. Second, Indigenous, and in particular Aboriginal, people are over-represented in custody. Third, Indigenous people are at a higher risk for suicide in the general community. Moreover, suicide is a leading cause of death in custodial settings, and prisoners are at a high risk for mortality on release from custody. An understanding of the predictors of suicidal ideation and suicide attempts for Indigenous people in custody is therefore overdue to help assist custodial and post-release mental health services detect and manage at-risk clients.

### Prevalence of suicidal behaviours

Findings indicated that the majority of study participants with available data had engaged in suicidal behaviours. Approximately 64% of participants reported lifetime ideation and approximately 28% reported ideation during the past 12 months. More than half the sample (54.5%) had attempted suicide. These proportions are higher than previous Australian studies with male Indigenous prisoners (from New South Wales and Queensland) which range from approximately 18% to 35% for lifetime ideation and 12% to 25% for lifetime attempts [9, 39, 40]. The discrepancy between the prevalence of suicidal behaviours among Indigenous males in custody in Victoria as documented in this study, and other jurisdictions are sizable. There may be methodological differences across studies (i.e., data collection approaches). This study comprised a wide-ranging and detailed interview that included cultural aspects and was conducted with an Indigenous well-being officer. It is possible that participants in this study felt particularly comfortable disclosing personal/sensitive information. A study from Western Australia which employed similar collection methods to this study, also discovered a higher than average prevalence of suicidal ideation and attempts for male and female Indigenous prisoners [45]. Although research from Queensland found much lower proportions of suicidal behaviours for male Indigenous prisoners using similar interview methods [46]. The higher proportions of suicidal behaviour in this study may also be a reflection of the poor health and social conditions experienced by some Indigenous males in Victoria, and particularly those who are justice-involved [47]. In line with prior research, the majority of participants who reported suicidal behaviours endured these experiences in the community [46]. Access to suitable mental health services/interventions in the community may also be limited for some Indigenous Victorians at risk for suicide in the community. Furthermore, correctional health care services may also be limited in their capacity to identify/manage/reduce risk factors for suicide for Indigenous people who frequently transition between custody and the community. The state of Victoria has one of the lowest imprisonment rates in the country and also hosts the smallest proportion of Indigenous prisoners of any Australian state or territory [26]. As such, Indigenous people in custody in Victoria may be more likely to possess numerous unmet health and social needs and pose a heightened risk for suicidal and offending behaviours.

### Correlates of suicidal behaviours

For participants in this study, none of the variables significantly predicted lifetime suicidal ideation, while bereavement was associated with more immediate ideation. The presence of ideation generally, predicted a suicide attempt along with a diagnosis of PTSD. Substance use disorder, custodial history or community location were not predictive of suicidal behaviours for the cohort.

The sole predictor of recent (12 month) suicidal ideation in the multivariate model was having a close friend or family member pass away over the past 12 months. Cycles of bereavement are commonplace in communities where there are a high numbers of premature and preventable deaths [13]. Grief in these circumstances may be particularly heightened due to the regularity of death, particularly of younger friends and family members, the rates at which the deaths are by suicide and the importance and meaning many Indigenous people afford to family/community connection [13]. For those who are incarcerated grief may be unresolved due to the lack of social support and distance from community during this time, prompting bouts of suicidal ideation.

There were no significant predictors of lifetime suicidal ideation in the model. However the odds of lifetime suicidal ideation occurring when a diagnosis of either PTSD or a psychotic disorder were present was particularly high compared to an absence of the disorders. Both PTSD [48, 49] and Psychosis [50] are commonly linked with suicidal behaviours in the general population. Moreover high rates of PTSD [51] and psychotic disorders [29, 40, 52] have been identified in correctional samples with PTSD/experiences of trauma associated with lifetime suicidal behaviours among prisoners [53, 54]. Rates of PTSD and links with suicidal behaviours are especially pronounced for Indigenous prisoners [55].

A diagnosis of PTSD was also one of two significant predictors of lifetime suicide attempts in this study, although this relationship was at the univariate level. High rates of violence (intimate partner violence, physical and sexual assault) have been reported in some Indigenous communities increasing the likelihood of experiencing various traumas. Indigenous prisoners’ lives are often punctuated with numerous traumatic events including violence exposure, separation from family and personal loss [56, 57]. Moreover, a body of literature on Historical Trauma describes the downstream collective impact of past colonial injustices such as state sanctioned child removal, land dispossession, social exclusion, discrimination and forced acculturation on subsequent Indigenous generations [4, 58–60]. Here, a personal sense of loss, grief and hopelessness is inextricably linked to collective experiences of trauma and despair [61]. While the nature and extent of the trauma witnessed by participants was unspecified in this study, its presence relative to its absence appears to significantly increase suicide risk. Indigenous male prisoners have been found to present with more traumatic symptomatology than non-Indigenous male prisoners [62]. The second, and strongest predictor of a suicide attempt was lifetime suicidal ideation. This finding was particularly salient. Almost three-quarters of participants reporting lifetime ideation had made a suicide attempt, indicating a pronounced link between these two actions in this sample. The ideation-attempt relationship has been established in prior forensic research, yet may be stronger in Indigenous populations [3]. This finding suggests that correctional institutions must make a concerted effort to carefully identify and monitor Indigenous clients who present with, or have histories of suicidal ideation.

Some caution is advised when generalising the findings to Indigenous men in custody in other regions of Australia, or women in custody as there are likely to be gendered issues for justice-involved men and women. Moreover, while the MINI has been administered to Indigenous populations in prior research, it is not known as to whether the assessment is culturally appropriate [63] or concordant with clinical diagnoses for such populations. It is also possible that mood and anxiety disorders may have been over-estimated by the MINI as has been found in prior research with general populations [43]. Estimates must also be considered in light of the small sample size. The present findings indicate considerable variability in confidence interval estimates, which is likely due to the low event per variable ratio in the current analysis [64]. Moreover the sample size did not allow for additional predictors to be added to the models. As such, we preferenced factors that we believed were most relevant in relation to suicidal behaviours. Further research should aim to identify which strength factors (i.e., resilience, cultural engagement, wellbeing etc.) lower the risk of suicidal behaviours.

### Implications

The correlates of suicidal behaviours for Indigenous people in custody in Victoria likely manifest in the community, denoting a public health response. Such a response needs to be considered within an Aboriginal and Torres Strait Islander men’s health and wellbeing framework. Trauma and bereavement are key factors, and point to the various social challenges faced by many Indigenous people. Surveys of the general Australian Indigenous population have discovered high rates family stressors and trauma exposure [65, 66]. In addition, the death of close family members and mental health problems are reported by a large minority [65]. Prevention must therefore begin in communities where these issues are particularly widespread and suicidal behaviour is high. The development of low-intensity mental health service infrastructure in affected communities to promote awareness and provide accessible, culturally informed support and treatment is required. This may include least-restrictive evidence based interventions that have flexible delivery and dosage options and can be administered or overseen by a range of service professionals. Moreover, community-based wellbeing workers could be trained to recognize risk factors, provide immediate aid and then link the individual with clinical services [67, 68]. Assistance to family members and peers who have recently been affected by suicide should be a part of any program. Seasonal variations in service intensity should also be considered given that suicidal behaviours in some Indigenous communities are increased during the hotter months. Evidently, accessible mental health services are just one component of a broader set of socio-economic and community factors underpinning suicidal behaviours. It is likely that such regions also undergo social and economic exclusion community dysfunction, family conflict and frequent law-breaking behaviour. Indigenous prisoners may disproportionately reside in such settings, often described as ‘pathogenic’ neighbourhoods [68]. As such, many Indigenous males in this study may have arrived in custody with pre-existing, unmet mental health concerns and some with unresolved anger, frustration and grief.

Although disconnection from family and community and the rigors of prison life may exacerbate these pathologies, evidence suggests that most Indigenous prisoners experience improved mental health and self-care during periods of incarceration [69, 70]. Better access to mental health care, general health care, daily routine and structure, and opportunities for social and cultural activities perhaps contribute to this outcome [70–72]. Nonetheless, correctional institutions should continue to improve custodial suicide prevention and management initiatives. Prison presents an opportunity to address the needs of vulnerable at-risk clients through therapy, improving coping skills and providing opportunities for meaningful activities. Screening for suicide-risk and the regular monitoring of clients with histories of suicidal behaviours and/or current acute levels of distress are useful preventive mechanisms [33]. This requires that corrections staff are trained in mental health first aid and recognizing risk factors for suicidal behaviours. The evidence for an increased post-release suicide risk for Indigenous prisoners is equivocal. However, a return to a community with numerous social challenges and a communal high risk for suicide may increase the individual’s risk for suicide on release. It is important that the predictors of suicidal behaviours identified in this study (mood disorders, exposure to trauma, bereavement) and their antecedents are addressed prior to, during and after re-entry. A dearth of treatment options in these spaces, particularly when transitioning back to the community, will ensure a continued risk for suicidal behaviours and possibly re-offending.

## Conclusion

This study found that a high proportion of Indigenous men in custody in Victoria have experienced lifetime suicidal ideation and over one-half have attempted suicide. A smaller, though not insignificant number had experienced ideation over the past 12 months. Having a loved one pass away within the past 12 months predicted recent ideation. Lifetime ideation and a diagnosis of PTSD were significant predictors of a lifetime suicide attempt.

## Abbreviations

DSM-IV: Diagnostic and statistical manual of mental disorders – Fourth Edition
ICD-10: International classification of diseases and related health problems
KPMHS: Koori prisoner mental health and cognitive function study
MINI: Mini international neuropsychiatric interview

## References

[1] World Health Organization. Global Health Estimates 2016: Deaths by cause, age, sex, by country and by region, 2000-2016. http://www.who.int/healthinfo/global_burden_disease/estimates/en/. Accessed 15 Aug 2018.

[2] Australian Bureau of Statistics [ABS]. 3303.0 – Causes of Death, Australia, 2016. 2017. http://www.abs.gov.au/ausstats/abs@.nsf/0/B86F926987EE114CCA257E19000B92CB?Opendocument. Accessed 15 Nov 2017.

[3] Armstrong G, Pirkis J, Arabena K, Currier D, Spittal MJ, Jorm AF (2017). Suicidal behaviour in indigenous compared to non-indigenous males in urban and regional Australia: prevalence data suggest disparities increase across age groups. Aust N Z J Psychiatry.

[4] Atkinson J (2002). Trauma trails: recreating song lines.

[5] Dudgeon P, Calma T, Holland C (2017). The context and causes of the suicide of indigenous people in Australia. J Indigenous Wellbeing.

[6] Hunter E, Harvey D (2002). Indigenous suicide in Australia, New Zealand, Canada, and the United States. Emerg Med J.

[7] Hunter E, Milroy H (2006). Aboriginal and Torres Strait islander suicide in context. Arch Suicide Res.

[8] Johnston E (1991). Final report of the Royal Commission into aboriginal deaths in custody.

[9] Larney S, Topp L, Indig D, O’Driscoll C, Greenberg D. A cross-sectional survey of prevalence and correlates of suicidal ideation and suicide attempts among prisoners in New South Wales, Australia. BMC Public Health. 2012;12. 10.1186/1471-2458-12-14 PMC3276432 22225627

[10] Priest N, Paradies YC, Gunthorpe W, Cairney SJ, Sayers SM (2011). Racism as a determinant of social and emotional wellbeing for aboriginal Australian youth. Med J Aust.

[11] Procter NG (2005). Parasuicide, self-harm and suicide in aboriginal people in rural Australia: a review of the literature with implications for mental health nursing practice. Int J Nurs Pract.

[12] Ralph N, Hamaguchi K, Cox M (2006). Transgenerational trauma, suicide and healing from sexual abuse in the Kimberley region. Aust Pimatisiwin.

[13] Silburn S, Robinson G, Leckning B, Henry D, Cox A, Kickett D, Dudgeon P, Milroy H, Walker R (2014). Preventing suicide among aboriginal Australians. Working together: aboriginal and Torres Strait islander mental health and wellbeing principles and practice.

[14] Stewart LM, Henderson CJ, Hobbs MST, Ridout SC, Knuiman MW (2003). Risk of death in prisoners after release from jail. Aust N Z J Public Health.

[15] Tatz C (2005). Aboriginal suicide is different: a portrait of life and self-destruction.

[16] ATISISPEP. The Aboriginal and Torres Strait Islander Suicide Prevention Evaluation Project – Kimberly Roundtable Report. 2015: http://www.atsispep.sis.uwa.edu.au/__data/assets/pdf_file/0009/2862603/Kimberley-Roundtable-Report-Final-March.pdf. Accessed 20 Dec 2017.

[17] Calabria B, Doran CM, Vos T, Shakeshaft AP, Hall W (2010). Epidemiology of alcohol-related burden of disease among indigenous Australians. Aust N Z J Public Health.

[18] Commonwealth of Australia. Alcohol, hurting people and harming communities*.* 2015. https://www.aph.gov.au/Parliamentary_Business/Committees/House/Indigenous_Affairs/Alcohol/Report. Accessed 5 Dec 2017.

[19] Measey MA, Li SQ, Parker R, Wang Z (2006). Suicide in the Northern Territory, 1981-2002. Med J Aust.

[20] Moore E, Gaskin C, Indig D (2015). Attempted suicide, self-harm, and psychological disorder among young offenders in custody. J Correct Health Care.

[21] Soole R, Kolves K, De Leo D (2014). Suicides in aboriginal and Torres Strait islander children: analysis of Queensland suicide register. Aust N Z J Public Health.

[22] Gee G, Dudgeon P, Schultz C, Hart A, Kelly K, Dudgeon P, Milroy H, Walker R (2014). Aboriginal and Torres Strait islander social and emotional wellbeing. Working together: aboriginal and Torres Strait islander mental health and wellbeing principles and practice.

[23] Shepherd SM, Adams Y, McEntyre E, Walker R (2014). Violence risk assessment in Australian aboriginal offender populations – a review of the literature. Psychol Public Policy Law.

[24] Wundersitz J. Indigenous perpetrators of violence: prevalence and risk factors for offending. Research and public policy series, 105. 2010. Accessed from: https://aic.gov.au/publications/rpp/rpp105. Accessed 30 Sept 2018.

[25] Productivity Commission (2011). Steering Committee for the Review of government service provision. Overcoming indigenous disadvantage: key indicators 2011.

[26] Australian Bureau of Statistics [ABS]. 4517.0 – Prisoners in Australia*,* 2017. 2017. http://www.abs.gov.au/ausstats/abs@.nsf/Lookup/by%20Subject/4517.0~2017~Main%20Features~Overview~3. Accessed 21 Nov 2017.

[27] Fazel Seena, Ramesh Taanvi, Hawton Keith (2017). Suicide in prisons: an international study of prevalence and contributory factors. The Lancet Psychiatry.

[28] Fazel S, Bains P, Doll H (2006). Substance abuse and dependence in prisoners: a systematic review. Addiction.

[29] Fazel S, Seewald K (2012). Severe mental illness in 33588 prisoners worldwide: systematic review and meta-regression analysis. Br J Psychiatry.

[30] Fazel S, Cartwright J, Norman-Nott A, Hawton K (2008). Suicide in prisoners: a systematic review of risk factors. J Clin Psychiatry.

[31] Kimonis ER, Skeem JL, Edens JF, Douglas KS, Lilienfeld SO, Poythress NG (2010). Suicidal and criminal behavior among female offenders: the role of abuse and psychopathology. J Personal Disord.

[32] Webster CD, Douglas KS, Eaves D, Hart SD, Webster CD, Jackson MA (1997). Assessing risk of violence to others. Impulsivity: theory, assessment, and treatment.

[33] World Health Organization. Preventing suicide in jails and prisons. 2007. Accessed from: http://www.who.int/mental_health/prevention/suicide/resource_jails_prisons.pdf. Accessed 28 Dec 2017.

[34] Baker A, Cussen T. Deaths in custody in Australia: National deaths in custody program 2011–12 and 2012–13. AIC Monitoring Reports 26. 2015. Accessed from: https://aic.gov.au/publications/mr/mr26. Accessed 30 Sept 2018.

[35] Kariminia A, Law MG, Butler TG, Levy MH, Corben SP, Kaldor JM, Grant L (2007). Suicide risk among recently released prisoners in New South Wales, Australia. Med J Aust.

[36] Pratt D, Piper M, Appleby L, Webb R, Shaw J (2006). Suicide in recently released prisoners: a population-based cohort study. Lancet.

[37] Spittal MJ, Forsyth S, Pirkis J, Alati R, Kinner SA (2014). Suicide in adults released from prison in Queensland, Australia: a cohort study. J Epidemiol Community Health.

[38] Willis M, Baker A, Cussen T, Patterson E. Self-inflicted deaths in Australian prisons. Trends & issues in crime and criminal justice (No. 513). 2016. Accessed from: https://aic.gov.au/publications/tandi/tandi513. Accessed 30 Sept 2018.

[39] Grigg K, Ogloff JRP. Considerations for suicide prevention in Australia’s prisoners. InPsych. 2016;38(1). https://www.psychology.org.au/inpsych/2016/feb/grigg. Accessed 15 Jan 2018.

[40] Indig D, Topp L, Ross B, Mamoon H, Border B, Kumar S, McNamara M (2010). 2009 NSW inmate health survey: key findings report.

[41] Indig D, McEntyre E, Page J, Ross B (2010). 2009 NSW inmate health survey: aboriginal health report.

[42] Sheehan DV, Lecrubier Y, Sheehan KH, Amorim P, Janavs J, Weiller E, Hergueta T, Baker R, Dunbar GC (1998). The Mini-international neuropsychiatric interview (M.I.N.I): the development and validation of a structured diagnostic psychiatric interview for DSM-IV and ICD-10. J Clin Psychiatry.

[43] Verhoeven FEA, Swaab LSMA, Carlier IVE, van Hemert AM, Zitman FG, Ruhe HG, Schoevers RA, Giltay EJ (2017). Agreement between clinical and MINI diagnoses in outpatients with mood and anxiety disorders. J Affect Disord.

[44] Clough AR, D’Abbs P, Cairney S, Gray D, Maruff P, Parker R, O’Reilly B (2005). Adverse mental health effects of cannabis use in two indigenous communities in Arnhem Land, Northern Territory, Australia: exploratory study. Aust N Z J Psychiatry.

[45] Fleming J, Gately N, Kraemer S (2011). Creating HoPE: mental health in Western Australian maximum security prisons. Psychiatry Psychol Law.

[46] Queensland Government (2009). Inside out —the mental health of aboriginal and Torres Strait islander people in custody report.

[47] Halacas C, Adams K (2015). Keeping our mob healthy in and out of prison: exploring prison health in Victoria to improve quality, culturally appropriate health care for aboriginal people.

[48] Sareen J, Houlahan T, Cox B, Asmundson G (2005). Anxiety disorders associated with suicidal ideation and suicide attempts in the national comorbidity survey. J Nerv Ment Dis.

[49] Krysinska K, Lester D (2010). Post-traumatic stress disorder and suicide risk: a systematic review. Arch Suicide Res.

[50] Bromet EJ, Nock MK, Saha S, Lim CCW, Aguilar-Gaxio-la S, Al-Hamzawi A (2017). Association between psychotic experiences and subsequent suicidal thoughts and behaviors. JAMA Psychiatry.

[51] Goff A, Rose E, Rose S, Purves D (2007). Does PTSD occur in sentenced prison populations? A systematic literature review. Crim Behav Ment Health.

[52] Butler T, Andrews G, Allnutt S, Sakashita C, Smith NE, Basson J (2006). Mental disorders in Australian prisoners: a comparison with a community sample. Aust N Z J Psychiatry.

[53] Shepherd S, Spivak B, Borschmann R, Kinner SA, Hachtel H (2018). Correlates of self-harm and suicide attempts in justice-involved young people. PLoS One.

[54] Kenny DT, Lennings CJ, Munn OA (2008). Risk factors for self-herm and suicide in incarcerated young offenders: implications for policy and practice. J Forensic Psychol Pract.

[55] Heffernan E, Andersen K, Davidson F, Kinner SA (2015). PTSD among aboriginal and Torres Strait islander people in custody in Australia: prevalence and correlates. J Trauma Stress.

[56] Day A, Davey L, Wanganeen R, Howells K, DeSantolo J, Nakata M (2006). The meaning of anger for Australian indigenous offenders. Int J Offender Ther Comp Criminol.

[57] Honorato B, Caltabiano N, Clough AR (2016). From trauma to incarceration: exploring the trajectory in a qualitative study in male prison inmates from North Queensland, Australia. Health Justice..

[58] Czyzewski K. Colonialism as a broader social determinant of health. Int Indigenous Policy J. 2011;2:1–14.

[59] Paradies Y (2016). Colonisation, racism and indigenous health. J Popul Res.

[60] Sherwood J (2013). Colonisation – it’s bad for your health: the context of aboriginal health. Contemp Nurse.

[61] Lawson-Te Aho K, Liu JH (2010). Indigenous suicide and colonization: the legacy of violence and the necessity of self-determination. Int J Confl Violence.

[62] Day A, Davey L, Wanganeen R, Casey S, Howells K, Nakata M (2008). Symptoms of trauma, perceptions of discrimination, and anger. A comparison between Australian indigenous and nonindigenous prisoners. J Interpers Violence.

[63] Hackett M, Farnbach S, Gallagher M, Skinner T, Teixeira-Pinto A, Askew D, Gee G, Cass A, Brown A (2016). Getting it right: study protocol to determine the diagnostic accuracy of a culturally-specific measure to screen for depression in aboriginal and/or Torres Strait islander people. BMJ Open.

[64] Greenland S, Mansournia MA, Altman DG (2016). Sparse data bias: a problem hiding in plain sight. BMJ.

[65] Australian Bureau of Statistics [ABS]. 4727.0.55.001 – Australian Aboriginal and Torres Strait Islander Health Survey: First Results, Australia, 2012–13*.* 2015. Accessed from: http://www.abs.gov.au/ausstats/abs@.nsf/0/C0E1AC36B1E28917CA257C2F001456E3?opendocument.

[66] Nadew GT (2012). Exposure to traumatic events, prevalence of posttraumatic stress disorder and alcohol abuse in aboriginal communities. Rural Remote Health.

[67] Australian Government. National Aboriginal and Torres Strait Islander Suicide Prevention Strategy. 2013. Accessed from: http://www.health.gov.au/internet/main/publishing.nsf/content/mental-pub-atsi-suicide-prevention-strategy. Accessed 10 Nov 2017.

[68] Closing the Gap Clearinghouse (2013). Strategies to minimise the incidence of suicide and suicidal behaviour (Resource sheet No. 18).

[69] Australian Institute of Health and Welfare (2015). The health of Australia’s prisoners 2015.

[70] Shepherd SM, Ogloff JRP, Thomas SDM (2016). Are Australian prisons meeting the needs of indigenous prisoners?. Health Justice.

[71] Lafferty L, Treloar C, Chambers GM, Butler T, Guthrie J (2016). Contextualising the social capital of Australian aboriginal and non-aboriginal men in prison. Soc Sci Med.

[72] Shepherd SM, Delgado RH, Sherwood J, Paradies Y. The impact of indigenous cultural identity and cultural engagement on violent offending. BMC Public Health. 2018;18. 10.1186/s12889-017-4603-2 PMC5525355 28738789

